# The Evidence for Efficacy of Osteoporosis Treatment in Men with Primary Osteoporosis: A Systematic Review and Meta-Analysis of Antiresorptive and Anabolic Treatment in Men

**DOI:** 10.4061/2011/259818

**Published:** 2011-06-24

**Authors:** Peter Schwarz, Niklas Rye Jorgensen, Leif Mosekilde, Peter Vestergaard

**Affiliations:** ^1^Research Center of Aging and Osteoporosis, Department of Medicine, Glostrup Hospital, 2600 Glostrup, Denmark; ^2^Faculty of Health Science, Copenhagen University, Copenhagen, Denmark; ^3^Department of Clinical Biochemistry, Glostrup Hospital, 2600 Glostrup, Denmark; ^4^Department of Endocrinology and Internal Medicine, MEA, THG, Aarhus University Hospital, Denmark

## Abstract

* Purpose*. Fragility fractures in men constitute a major worldwide public health problem with a life-time risk of 13%. It cannot be directly inferred that antiosteoporotic drugs effective in women have the same effect in men. Our aim was to appraise the existing evidence for efficacy of osteoporosis treatment in men. *Methods*. This study was a systematic review of the published literature on the clinical efficacy of medical osteoporosis therapy in the reduction of fracture risk in men (age *> *50 years). Studies included were randomised, placebo-controlled trials of men. *Results*. Five BMD studies of antiresorptive treatment were included. All studies showed an increase in BMD, but there was only a nonsignificant trend in the reduction of clinical fractures. Three BMD studies of anabolic treatment with teriparatide were also included. These showed a significant mean increase in spine BMD and for vertebral fractures a non-significant trend towards a reduction was seen. *Conclusion*. The evidence of medical osteoporosis treatment in men is scant and inconclusive due to the lack of prospective RCT studies with fracture prevention as primary end point. So far, all evidence is based on BMD increases in small RCT studies showing BMD increases comparable to those reported in postmenopausal women.

## 1. Introduction

Fragility fractures in men constitute a major worldwide public health problem [1] although the incidence and gender ratio varies between countries [2]. The life-time risk of any fracture in the hip spine or distal forearm in men aged >50 years has been estimated to be 13% compared with 40% in females [3] The fractures occur 5–10 years later in men than in women [4], but the increasing longevity in men is likely to increase the public health burden of the fractures [2]. Follow-up studies, including the osteoporotic fractures in men (MrOS) cohort, have established that 1 SD deviation in areal bone mineral density (aBMD) equally predict fracture risk for spine and hip in men and in women [2, 5]. Therefore, the lower incidence of fractures in males compared with females in all probability reflects that at any, age fewer males than women have compromised biomechanical competence because of smaller bones, lower volumetric BMD (vBMD), thinner cortices, thinner trabeculae, microfractures with disruption of trabecular structure, or higher bone turnover [2]. Moreover, the etiology differs between males and females. Hypogonadism is a risk factor for osteoporosis in both sexes, but the prevalence and progression of sexhormone deficiency differs. Testosterone deficiency is a risk factor for male osteoporosis, whereas estradiol deficiency is a triggering factor in both sexes. Furthermore, the influence of environmental factors like alcohol, smoking, and risk of falling may differ between sexes. Because of the described gender differences in risk factors, pathophysiology, and bone structure, it cannot be directly inferred that anabolic or antiresorptive drugs that prevent BMD loss and osteoporotic fractures in females [6–26] have the same effect in males. However, only few small randomized controlled trials (RCTs) on the treatment efficacy of antiosteoporotic drugs have been performed in men. It is, therefore, important to appraise the existing evidence of the impact of osteoporosis treatment in elderly and old men.

## 2. Objectives

This is a systematic review and meta-analysis of the published literature on RCT studies of clinical efficacy of antiresorptive and anabolic therapy in the reduction of fracture risk in elderly and old men. The following end points were used: RCT studies on vertebral fracture reduction, nonvertebral fracture reduction, and hip fracture reduction for men with primary osteoporosis.

## 3. Materials and Methods

### 3.1. Eligibility Criteria for Study Inclusion

Studies should be randomised placebo-controlled trials of at least 12 months duration (anti-resorptive treatment) or of at least 6 months duration (anabolic therapy). The antiresorptive medications included as exposure variables in the search were strontium ranelate, bisphosphonates, denosumab, and miacalcic. Strontium ranelate was here categorized as antiresorptive although there is growing evidence that it also may exert anabolic properties. The anabolic treatments included the truncated PTH(1–34) analog teriparatide and the full length PTH(1–84) preotact. Of the bisphosphonates, we included all commercially available medications for oral or intravenous treatment. That is, etidronate, ibandronate, risedronate, alendronate and zoledronate.

Only RCT studies where the primary end-points were vertebral, nonvertebral or hip fracture risk reductions, and/or BMD changes were included.

### 3.2. Search Methods

An electronic search of PubMed (1951 and onwards), Embase (1974 and onwards), Science Citation Index (1945 and onwards), and the Cochrane Central Register of Controlled Trials was performed. The search date was December 19, 2010. 

Abstracts of all possibly relevant articles were reviewed for potential eligibility (assessed by P.Schwarz and P. Vestergaard). Discrepancies were solved through discussion. Those deemed eligible and those that did not had adequate information to confirm their inclusion underwent a full text review. The retrieval was based on published papers only. We examined reference lists of retrieved studies for further relevant publications. If several publications were reported based on the same trial data we chose the report with the longest followup. Pooled analyses and subgroup analyses were not included due to their weak statistical value. No contacts were made with lead authors or pharmaceutical companies.

The keywords producing the majority of results, that is “osteoporosis,” “treatment,” and “men” were chosen. This search gave 10.314 trials (Table 1). Subsequently, a search was made separately for each of the respective drugs. This method did not produce any articles with fracture reduction as end point in men, so the same search was repeated with BMD as a substitute endpoint for fracture risk reduction. Concerning antiresorptive treatment, this method produced 13 potential papers of which 7 reported open-labelled and/or not randomised studies, leaving 6 papers to be included. 

As to anabolic treatment, 5 potential papers were identified. However, one study only reported data with a mixture of men and women without the possibility of extracting data solely on men, leaving 4 papers for evaluation.

All data were summarised in a formula including number of patients, age, gender, BMI, BMD, duration, and main outcomes measured (Table 2).

### 3.3. Statistical Analyses

The meta-analysis was performed as a random effects model using the inverse of the standard deviation of the individual BMD and fracture risk parameters from each study as weights for the estimates as proposed by Böhning [33]. Tests for heterogeneity and publication bias were performed. *P* < 0.05 was considered statistically significant.

## 4. Results

### 4.1. Antiresorptive Drugs

Five antiresorptive drugs, alendronate (2 studies), risedronate (1 study), ibandronate (1 study), zoledronate (2 studies), and nasal miacalcic (1 study), have been investigated in male populations with osteoporosis (Table 2) [27–32, 34]. The study zoledronate study of Orwoll et al. [32] was excluded, as it was not placebo-controlled, and the zoledronate study of Lyles et al. [34] was a mixture of men and women, and data on men could not be extracted. The remaining five studies had BMD as their primary end-point (Table 3).

#### 4.1.1. Changes in BMD

Orwoll et al. [27] reported a significant increase in bone mineral density of 7.1 ± 0.3% at the lumbar spine, 2.5 ± 0.4% at the femoral neck, and 2.0 ± 0.2% for the total body (*P* < 0.001 for all comparisons with baseline). The increase in BMD in the alendronate group was greater than that in the placebo group at all measurement sites (Table 3, *P* < 0.001). In a 3-year RCT, Gonnelli et al. [28] reported an increase in lumbar spine BMD of 4.2% at year 1, 6.3% at year 2, and 8.8% at year 3. BMD at the femoral neck and total hip increased 2.1% and 1.6%, respectively, at year 1, 3.2% and 2.9% at year 2, and 4.2% and 3.9% at year 3. In a 2-year RCT Boonen et al. [29] reported that treatment with risedronate resulted in a significant 4.5% (95% CI: 3.5–5.6%; *P* < 0.001) increase in lumbar spine BMD compared with placebo. In a 1-year RCT study, Orwoll et al. [30] reported an increase in lumbar spine BMD of 3.5%  (*P* < 0.001). BMD at the total hip increased by 1.8%  (*P* < 0.001) and femoral neck 1.2%  (*P* < 0.012) [30]. Trovas et al. [31] performed a 12-month RCT with nasal miacalcic. The men who were treated with calcitonin had a mean increase in BMD of 7.1 ± 1.7% at the lumbar spine. The increase in lumbar BMD in the calcitonin group was significantly greater than that in the placebo group (*P* < 0.05).

#### 4.1.2. Changes in Risk of Fractures

Three studies reported fractures as secondary endpoints. All studies had included few patients with a low mean age, and they all had a relatively short duration of 12–36 months (Table 2). 

The studies of Orwoll et al. (alendronate) [27] and Boonen et al. (risedronate) [29] both reported incidences of vertebral fractures (Table 3). Orwoll et al. found a significant reduction (*P* = 0.02) in vertebral fractures determined by quantitative methods and no effect on non-vertebral fractures. Boonen et al. found 2 new vertebral fractures after 2 years each in the risedronate group. There was a nonsignificant trend towards a reduction in all fractures (placebo 6 patients (6.5%); risedronate 9 patients (4.7%)).

### 4.2. Anabolic Drugs

Five studies were available on anabolic treatment with teriparatide in men [9, 35–38]. However, the study of Finkelstein et al. was not placebo controlled and therefore excluded [36], and the study of Kaufman et al. was based on the same men as reported in the study of Orwoll et al. [9] and therefore excluded as well. In addition, a newly published study report on both Japanese men and women was available [37]. However, data on men cannot be extracted from this publication and the included numbers of men were low (5 in the placebo group and 9 in the treatment group), this study was excluded as well [37]. No studies in men were available for preotact or any other anabolic medication. In all three included papers, the primary end point was BMD (Table 2).

#### 4.2.1. Changes in BMD

Compared with placebo Orwoll et al. [9] found a significant increase in lumbar spine (*P* < 0.001) and femoral neck (*P* = 0.029) BMD in the group receiving 20 *μ*g/day of teriparatide (Table 3). In the 40 *μ*g/day group, the increase in BMD compared with placebo was significant at the lumbar spine (*P* < 0.001), the total hip (*P* < 0.001), and the femoral neck (*P* < 0.001). The increase was higher in the 40 *μ*g/day than in the 20 *μ*g/day group at the lumbar spine (*P* < 0.001), the total hip (*P* = 0.009) and the femoral neck (*P* = 0.023). In the PTH-treated group, Kurland et al. [35] found a gain in lumbar spine BMD at 18 months of 13.5 ± 3.0% (*P* < 0.001 compared with placebo), whereas the increase in the femoral neck was 2.9 ± 1.5% (*P* < 0.05) (Table 3). 

**Figure 1:**
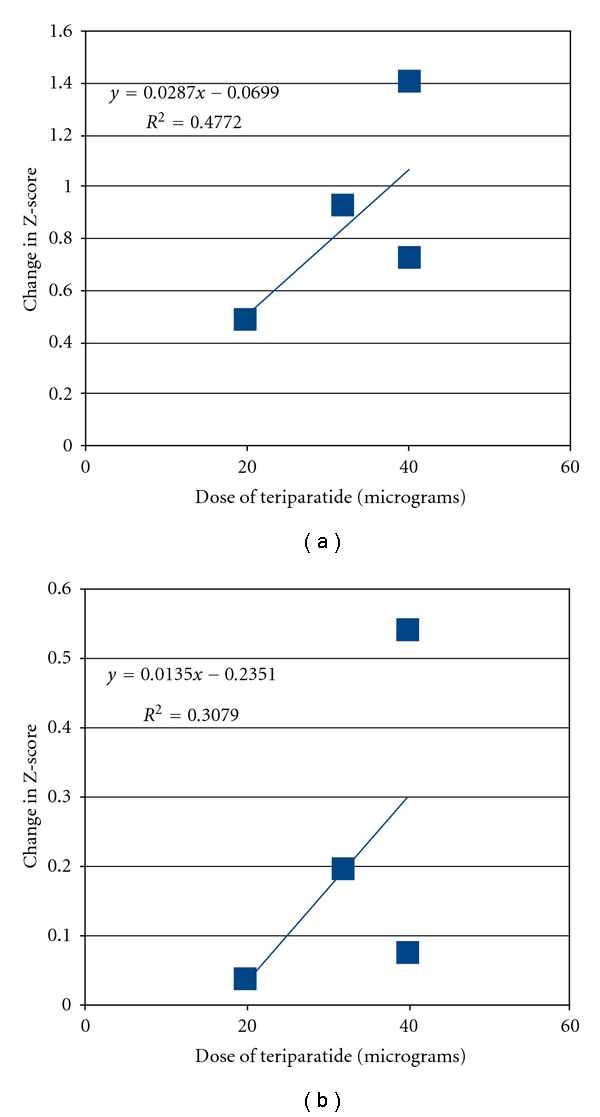
Increase in spine (a) and femur (b) BMD in the Teriparatide studies by daily dose.

The mean increase in BMD in all studies (*n* = 3) and subgroups (*n* = 4 in 3 studies) combined was 0.58 ± 0.02, *P* < 0.01 for spine BMD Z-score and 0.05 ±  0.01, *P* < 0.01 for femoral neck Z-score (Figure 1). 

#### 4.2.2. Changes in Risk of Fractures

Orwoll et al. [9] reported non-vertebral fractures as side effects in 6 patients (3 among 147 placebo treated, 2 among 151 treated with 20 micrograms of teriparatide, and 1 among 139 treated with 40 micrograms of teriparatide). Kurland et al. [35] reported data on the incidence of vertebral fractures (1 new fracture) among 6 PTH treated and 2 patients among 12 placebo-treated had new vertebral fractures (one and three new fractures, resp.). In average, the studies of Orwoll et al. and Kurland et al. yielded a reduction in risk of vertebral fractures of  RR = 0.60, 95% CI: 0.29–1.22, *P* for heterogeneity 0.71.

#### 4.2.3. Adverse Events

Focusing on adverse events in the anti-resorptive treatment group, the study of Orwoll et al. [27] showed that the incidence of overall GI adverse events was higher in the placebo group compared with the risedronate group (18% versus 8%). Also, withdrawal from the study because of adverse events was more frequent in patients taking placebo (9.7% versus 3.7%) [29]. For alendronate [27, 28], the results resemble the results in women. In the miacalcic study [31], no specific data are reported (Table 4). Among the anabolic studies, Orwoll et al. [9] reported 2 deaths in the teriparatide 20 *μ*g group. None of these was considered related to study drug or procedures. Three cancers occurred in the placebo group, three in the teriparatide 20 *μ*g group and none in Teriparatide 40 *μ*g group. There were no cases of osteosarcomas. In the two studies, it was concluded that the medication was well tolerated [9, 35].

## 5. Discussion

There is evidence that both antiresorptive and anabolic treatment compared with placebo increase BMD in osteoporotic males. However, fracture data in men are scant at all sites (vertebral, non-vertebral, and hip fractures), and there are no RCTs that evaluate antiresorptive or anabolic osteoporosis treatment in men with fractures as primary end point. Furthermore, studies with fractures as secondary end points are inconclusive. As a consequence, there is at present no well-established documented treatment for idiopathic osteoporosis in men. However, the fact that one in five men aged ≥50 years will suffer an osteoporotic fracture during their lifetime underscore the necessity to appraise the antifracture efficacy of various treatment modalities in men. 

The strength of this study is the systematic inclusion of all studies available in men receiving anti-resorptive treatment as well as anabolic osteoporosis treatments.

The limitations are the very low number of studies included in the meta-regression makes the evidence based on the method limited. Not only are the number of studies limited and the follow-up time short, the power of the studies to reveal significant effects on fracture risk is also low because of the limited number of patients included. Due to this we are not able to definitely conclude if one medication is in favor of others among men with primary osteoporosis.

In conclusion, the evidence of medical osteoporosis treatment in men is scant at all sites and inconclusive due to the lack of prospective large RCT studies with fracture prevention as primary endpoint. All evidence so far is based on BMD findings in small RCT studies showing increases comparable to those observed in studies in postmenopausal women.

## Figures and Tables

**Table 1:** Identifying key words.

Osteoporosis AND Treatment AND Men	10.314
AND alendronate	495
AND risedronate	215
AND ibandronate	63
AND didronate	300
AND zoledronic acid	127
AND strontium ranelate	50
AND denosumab	28
AND miacalcic	81
AND teriparatide	175
AND PTH(1–84)	17
AND preotact	1

**Table 2:** Baseline characteristics of included studies.

Study	Intervention (plus calcium and/or vitamin D)		Number of patients	Age (±SD)	BMI	BMD (lumbar; total hip; femoral neck) in g/cm^2^	BMD (lumbar; total hip; femoral neck) T score	Duration (months)	Outcomes measured	Lost to follow-up (intervention versus control)
Alendronate										

Orwoll et al. [27]	Placebo or 10 mg/d of alendronate (500 mg/d and 400–450 IU/d)	Placebo Alendronate	95 146	63 (12) 63 (13)	25 (3) 25 (3)		2.2; 2.1; 2.3 2.0; 2.1; 2.2	24	BMD	17% versus 14%
									Vertebral fractures. nonvertebral fractures (secondary endpoints)	

Gonnelli et al. [28]	Placebo (calcium) or 10 mg/d of alendronate (1000 mg/d)	Placebo	38	56.6 (10.4)	24.3 (2.9)	0.737 (0.103); 0.770 (0.099); 0.632 (0.100)		36	BMD. QUS	6 versus 7
		Alendronate	39	57.2 (9.9)	24.9 (2.4)	0.725 (0.110); 0.762 (0.101); 0.622 (0.090)				

Risedronate										

Boonen et al. [29]	Placebo or 35 mg of risedronate (1000 mg/d and 450–500 IU/d)	Placebo	93	62 (11)	25 (4)	0.824 (0.96);	−3.1 (0.9);	24	Lumbar spine BMD BMD at other sites. new vertebral fractures. clinical fractures (secondary endpoints)	16 versus 18
						0.763 (0.106); NA	−2.0 (0.7); NA			
		Risedronate	191	60 (11)	25 (4)	0.809 (0.99);	−3.3 (0.9);			
						0.768 (0.111); NA	−2.0 (0.7); NA			

Ibandronate										

Orwoll et al. [30]	Placebo or 150 mg Ibandronate/month	Placebo	47	65.0 (10.6)	24.8 (3.4)		−2.1 (0.68)	12	BMD	1
							−1.8 (0.70)			
							−2.3 (0.55)			
		Ibandronat	85	63.9 (11.2)	25.9 (4.1)		−2.1 (0.61)			3
							−1.7 (0.68)			
							−2.2 (0.50)			

Miacalcic										

Trovas et al. [31]	Placebo or 200 IU/d of miacalcic (1000 mg/d)	Placebo	13	51.6 (10.5)	25.7 (3.1)	0.847		12	BMD	0
						(0.190); NA;				
						0.753 (0.162)				
		Miacalcic	15	53.3 (13.7)	26.1 (2.4)	0.866			Vertebral fractures	
						(0.124); NA;				
						0.737 (0.116)				

Teriparatide										

Trovas et al. [31]	Placebo or 20 or 40 *μ*g Teriparatide/d versus Placebo	Placebo	147	59 (13)	25 (4)	0.85 (0.14)	−2.4 (1.2)	11	BMD	24
							−1.9 (0.8)			
							−2.7 (0.8)			
		Teriparatide 20 *μ*g/d	151	59 (13)	25 (4)	0.89 (0.15)	−2.0 (1.3)			11
							−1.8 (0.8)			
							−2.6 (0.8)			
		Teriparatide 40 *μ*g/d	139	58 (13)	25 (4)	0.87 (0.14)	−2.2 (1.2)			20
							−1.9 (0.9)			
							−2.7 (0.8)			

Orwoll et al. [32]	Placebo versus Teriparatide 32 *μ*g	Placebo	10	54.5 (2.6)	25.9 (1.5)	0.746 (0.03)	−3.3 (0.3)	18	BMD	0
						0.781 (0.02)	−1.7 (0.2)			
						0.650 (0.03)	−2.0 (0.2)			
		Teriparatide	13	49.5 (2.9)	24.3 (1.0)	0.731 (0.03)	−3.5 (0.2)			0
						0.774 (0.03)	−1.7 (0.2)			
						0.644 (0.02)	−1.9 (0.2)			

Fx, number of patients with one or more fractures.

**Table 3:** Anti-fracture effects.

Study	Vertebral fractures				Nonvertebral fractures				HIP fractures				BMD (lumbar; total hip; femoral neck)
	Treatment	*N* (%)	RR (95% CI)	NNT	Treatment	*N* (%)/*n *	RR (95% CI)	NNT	Treatment	*N* (%)	RR (95% CI)	NNT	Mean difference % (95% CI) or Percent change (SD)
Alendronate													

Orwoll et al. [27]	Placebo	NA (7.1)			Placebo	5 (5.3)							5.3 (4.3–6.3); 2.6 (1.5–3.7); 2.6 (1.5–3.7)*
	Alendronate	NA (0.8)			Alendronate	6 (4.1)							
Gonnelli et al. [28]	Placebo	NA											10; 4.2; 5.4*
	Alendronate	NA											

Risedronate													

Boonen et al. [29]	Placebo	0											4.5 (3.5–5.6)*; results for total hip and femoral neck in figures
	Risedronate	2											

Ibandronate													

Orwoll et al. [30]	Placebo	2			Placebo	0							NA
	Ibandronate	1			Ibandronate	2							

Miacakcic													

Trovas et al. [31]	Placebo	2											2.47; −0.68; NA
	Miacalcic	1											7.13; 0.41; NA

Teriparatide													

Trovas et al. [31]	Placebo	0			Placebo	3							0.52 (3.90) 0.54 (2.70) 0.31 (4.10)
							
							
	Teriparatide 20 *μ*g	0			Teriparatide 20 *μ*g	2							5.87 (4.50)*** 1.17 (2.94) 1.53 (3.95)**
							
	Teriparatide 40 *μ*g	0			Teriparatide 40 *μ*g	1							9.03 (6.46)*** 2.33 (4.41)*** 2.93 (6.34)***
							
							
							

Orwoll et al. [32]	Teriparatide	1 (17)				0							13.5 (3.0)*** NA
	Placebo	2 (17)				0							2.9 (1.5)**

*Difference between groups (95% CI); (*P* < .001 in favour of active treatment).

**Difference between groups (SD); *P* < .05 in favour of active treatment.

***Difference between groups (SD); *P* < .001 in favour of active treatment.

NA: not available.

**Table 4:** Adverse events.

Study	****	General (*N* (%))					Specific (*N* (%))		
	****	Any AE	Any serious AE	Death	Withdrawals due to AE				
Alendronate	****	****	****	****	****	****	Placebo	Alendronate	

Orwollet al. [27]	Placebo	****	22 (23)	****	10 (11)	Any upper GI AE	21 (22)	37 (25)	
	Alendronate	****	27 (18)	****	4 (3)	Dyspepsia	1 (1)	9 (6)	
						Abdominal pain	4 (4)	12 (8)	
						Esophagitis	1 (1)	1 (1)	
						Reflux	5 (5)	7 (5)	
Gonnelli et al. [28]	Placebo Alendronate	****		NA	****	****	NA	****	

Risedronate	****	****	****	****	****	****	Placebo	Risedronate	

Boonen et al. [29]	Placebo (*n* = 93)	68 (73)	15 (16)	3 (3)	9 (9.7)	Any upper GI AE	17 (18)	16 (8)	
						Constipation	5 (5)	16 (8)	
	Risedronate (*n* = 191)	134 (70)	29 (15)	2 (1)	7 (3.7)	Back pain	2 (2)	13 (7)	
						Arthralgia	8 (9)	11 (6)	
						Influenza	5 (5)	11 (6)	
						Nasopharyngitis	5 (5)	11 (6)	
						Headache	0 (0)	10 (5)	

Ibandronate	****	****	****	****	****	****	Placebo	Ibandronate	

Orwoll et al. [30]	Placebo	4 (9)	(7)	2	****	Any	4 (9)	16 (19)	
	Ibandronate	16 (19)	(9)	1	****	Abdominal pain	(0)	(3)	

Miacakcic	****	****	****	****	****				

Owroll et al. [30]	Placebo Miacalcic	Very well tolerated and no significant side effects	****	0	0	****	NA	****	

Teriparatide	****	****	****	****	****	****	Placebo	Teriparatide	Teriparatide

Trovas et al. [31]	Placebo	39 patients	3	0	7	Nausea Headache	3.4 NA	5.3 N A	18.7 10. 8
	Teriparatide 20 *μ*g	withdrew due to	3	2	14				
	Teriparatide 40 *μ*g	side-effects	0	0	18				
Orwoll et al. [32]	Teriparatide		0	0	0	Hypercalcemia	0	2	
	Placebo		0	0	0	Redness at inj. site	0	5	
